# The physical origin of rate promoting vibrations in enzymes revealed by structural rigidity

**DOI:** 10.1038/s41598-020-74439-5

**Published:** 2020-10-15

**Authors:** Yann Chalopin

**Affiliations:** Laboratoire EM2C, CNRS & CentraleSupelec, University of Paris-Saclay, 91190 Gif-sur-Yvette, France

**Keywords:** Biocatalysis, Protein function predictions, Biophysics, Network topology

## Abstract

Enzymes are the most efficient catalysts known to date. However, decades of research have failed to fully explain the catalytic power of enzymes, and most of the current attempts to uncloak the details of atomic motions at active sites remain incomplete. Here, a straightforward manner for understanding the interplay between the complex or irregular enzyme topology and dynamical effects at catalytic sites is introduced, by revealing how fast localized vibrations form spontaneously in the stiffest parts of the scaffold. While shedding light on a physical mechanism that allowed the selection of the picosecond (ps) timescale to increase the catalytic proficiency, this approach exposes the functional importance of localized motions as a by-product of the stability-function tradeoff in enzyme evolution. From this framework of analysis—directly accessible from available diffraction data—experimental strategies for engineering the catalytic rate in enzymatic proteins are proposed.

## Introduction

Proteins derive their tremendous range of biological functions from the intricate patterns of interactions between each other and with the myriad of other molecules in living organisms. These interrelations have been early understood from studies of the three-dimensional (3D) structures in complex with their binding partners-known as *lock and key*—that has given rise to several scientific advances. However, enzymes and, more generally proteins, are highly dynamical entities^1^. The knowledge of their structure’s dynamical nature is thus essential to establish a complete understanding of their functioning. From the search for catalytically optimal conformations to the transition over the barrier that defines the chemical step, protein kinematics occur over multiple timescales, ranging from ps to a few $\upmu$s^2,3^. Collective domain motions on the $\upmu$s–ms range have been identified and linked to several biochemical activities (see, for instance: hinge bending, allosteric changes, motor protein conformational changes^4^). In enzymatic proteins, however, faster atomic-scale fluctuations take place at active sites, influencing on reaction chemistry^2,5,6^. In a recent and controversial debate, these fast dynamical modes—termed rate promoting vibrations (RPV)—have been recognized as playing a key role in increasing the chemical rate in enzymes^7^. Several works have highlighted that RPVs correspond to localized energy that couples with the chemistry in the form of mechanical compressions between the enzyme residues and the reactant^7–10^. Active site residues have—on average—lower temperature factors than the non-active site ones. This implies that the former are in general stiffer than the latter^11,12^ and thus, active sites are subjected to higher cohesive forces^13–15^. This observation was first interpreted as functional improvement of the molecular structure through folding, as modulating the rigidity at the active site controls the thermal stability of enzymes^16–18^. Also, direct active site mutations have demonstrated to be a very efficient way of increasing protein stability^19^.

It is further interesting to note that the secondary and tertiary structure (folded) of enzymes is more conserved in evolution than the primary one (sequence)^20^. The backbone (BB) rigidity is a fundamental physical feature preserved by enzymes throughout evolution^21^. Consequently, it is commonly admitted that catalytic sites are energetically strained to maintain the stability-function tradeoff throughout evolution^22^, but is that all? This broad set of discoveries raises a central question that remains unexplored: What is the most basic physical or molecular mechanism that unifies the fast (i.e picosecond) dynamical properties with the emergence of an enzyme function (e.g., RPVs) and the conserved structural rigidity? As the flexibility or the disordered nature of enzymes is well understood in conformational changes, high-strained residues, which are often regarded as order, contain an undisclosed fundamental feature of the functioning of enzymes that emerges from an intermediate time scale between the change of shape and the crossing of the transition barrier.

## Materials and methods

### The dynamic of the backbone and wave propagation

**Figure 1:**
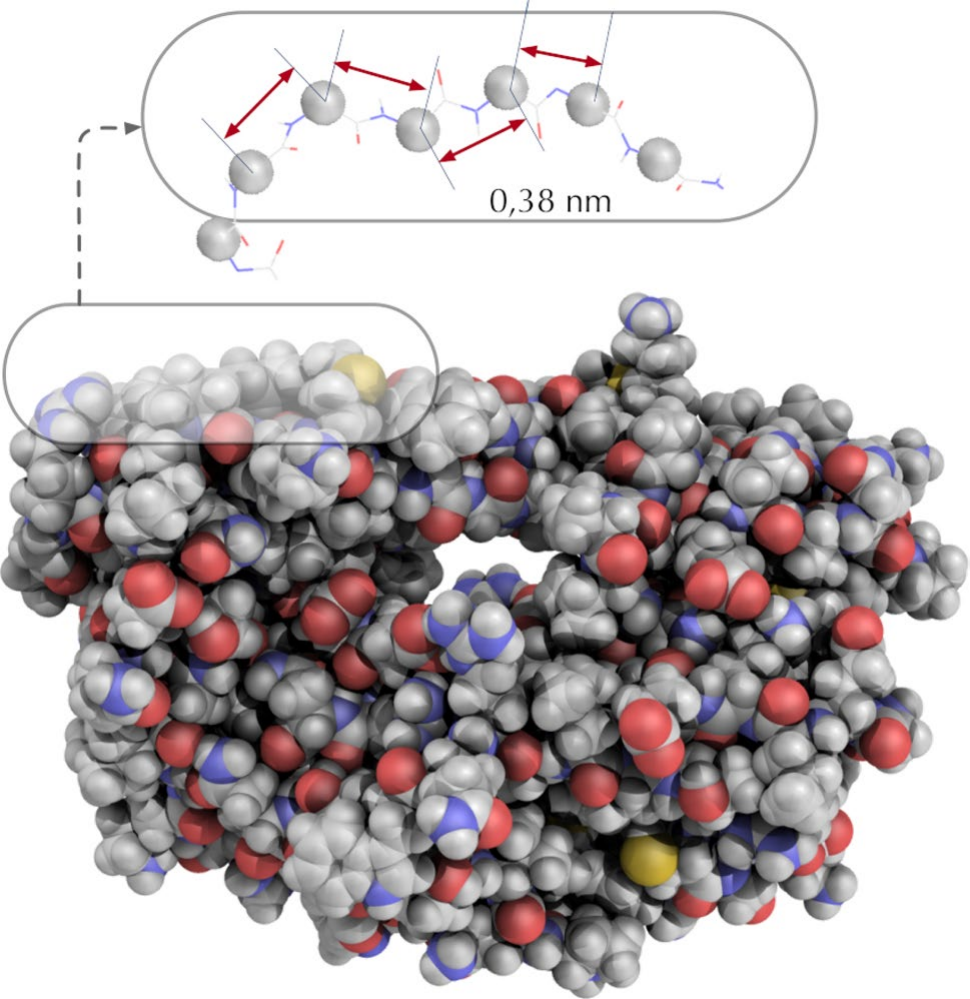
All atoms view of the HIV1-Protease (PDB id:1opw). The protein backbone is a periodic chain of $C\alpha$ carbon atoms separated by 3.8 Å. The figure was rendered using PyMOL (The PyMOL Molecular Graphics System; http://www.pymol.org).

In this work, enzymes are regarded as periodic chains of amino-acids (AA) with a period of 0.38 nm, as depicted in Fig. 1. This consideration is central in this work: The discrete translational symmetry along the AA sequence gives rise to wave propagation. In the case of enzymes, it consists of waves spreading and damping within the scaffold, an action constantly maintained by the environment temperature (Eq. (4)). The BB exhibits roughly $2\times {10^3}$ internal degrees of freedoms^23^ and consequently produce the same quantity of modes of ps timescales (typically 50 to 100 cm$^{-1}$). Hence, enzymes are regarded here as *fast* dynamical objects, made of a collection of randomly coupled harmonic oscillators. The hamiltonian of the molecule can be generally written as

1 $$\begin{aligned} H = \sum \limits _{i} E_i \end{aligned}$$

where the energy of each residue *i* is expressed as,

2 $$\begin{aligned} E_i=\frac{1}{2}\left\{ {{{{\dot{X}}}}_i^2} + \sum \limits _{j \sim i} {{\alpha _{ij}} ({{{X}}_i}-{{{X}}_j})^2}\right\} . \end{aligned}$$

*X* and respectively $\alpha$ stands for the displacement from equilibrium and the force constant respectively. The corresponding equations of motion is written as

3 $$\begin{aligned} {{{{\ddot{X}}}}_i} = - \sum \limits _{j \sim i} {{\alpha _{ij}}{{{X}}_i}} + \sum \limits _{j \sim i} {{\alpha _{ij}}{{{X}}_j}}. \end{aligned}$$

The force constant $\alpha$ are obtained from a parameter-free elastic network model (ENM) based on the work of Yang^24^.

The harmonic solutions of pulsation $\omega$ are expressed in a matrix form

4 $$\begin{aligned} {-\Delta _d}{} \mathbf{{X}} = {\omega ^2}{} \mathbf{{X}}. \end{aligned}$$

Here, $\omega ^2$ are the eigenvalues of $-\Delta _d$, an elliptic operator corresponding to the Laplacian on a graph. This operator is sometime called the dynamical matrix. The corresponding eigenvectors provide information on the amplitude of the normal modes (the phonons) of the protein backbone, while the eigenvalues $\omega ^2$ deliver information on the characteristic timescales $\tau$ of the vibrations :

5 $$\begin{aligned} \tau = 2\pi /\sqrt{(\omega ^2)} \end{aligned}$$

### Backbone rigidity: a central property

Rigidity and flexibility have a broad sense in protein science and engineering. These concepts—borrowed from mechanical engineering—are often regarded as a static attribute. Current research associates the rigidity of the protein scaffold with thermostability, but there is more to claim. A definition of the backbone rigidity is proposed here, derived from the internal molecular degree of freedom $X_i$. The local rigidity $R_i$ at each residue *i* is obtained from a second derivative of the energy of structure.

6 $$\begin{aligned} {R_i} = \frac{{{\partial ^2}{E_i}}}{{{\partial ^2}{X_i}}} , \end{aligned}$$

### Rigidity and temperature factor

The temperature factor, also termed B-factor (or the Debye–Waller factor), describes the attenuation of radiations (X-ray or neutron) induced by the thermal motions. The B-factors have been extensively exploited in a wide range of studies dealing with the importance of rigidity, flexibility, and domain motions, which—as mentioned in the introduction—are primordial in proteins in general. This quantity mirrors the atomic displacement fluctuations and delivers important information about internal dynamics. From the definition introduced above for the $C_\alpha$ atoms *i*, the beta factor is expressed as

7 $$\begin{aligned} {B_i} = 8{\pi ^2}\left\langle {X_i^2} \right\rangle /3. \end{aligned}$$

It is possible to introduce an alternative definition in Eq. (14) by replacing $\left\langle {X_i^2} \right\rangle$ by $\left\langle {{\bar{X}}_i^2} \right\rangle$, which corresponds to the Beta-factor obtained by averaging over the atoms instead of solely considering the $C_\alpha$.

### Localized vibrations and the backbone rigidity

From the concept of localization landscape introduced for electrons^25^, it has been reported that enzymes are subjected to the phenomena of wave localization^26^ that manifests itself as a striking correlation between the locations of active sites and the formation of thermal hot spots (vibrations). This central quantity is obtained by solving the linear system

8 $$\begin{aligned} - {{ \Delta }_h}u = (1) \end{aligned}$$

where

9 $$\begin{aligned} {{-\Delta _h}}:(\delta _{lm})={\left\{ \begin{array}{ll} c-\sum \limits _{m=1}^{N} {\alpha _{lm}} , &{} \hbox { if}\ l=m.\\ \alpha _{lm} {e^{ik({x_m} - {x_l})}} , &{} \text {otherwise}. \end{array}\right. } \end{aligned}$$

**Figure 2:**
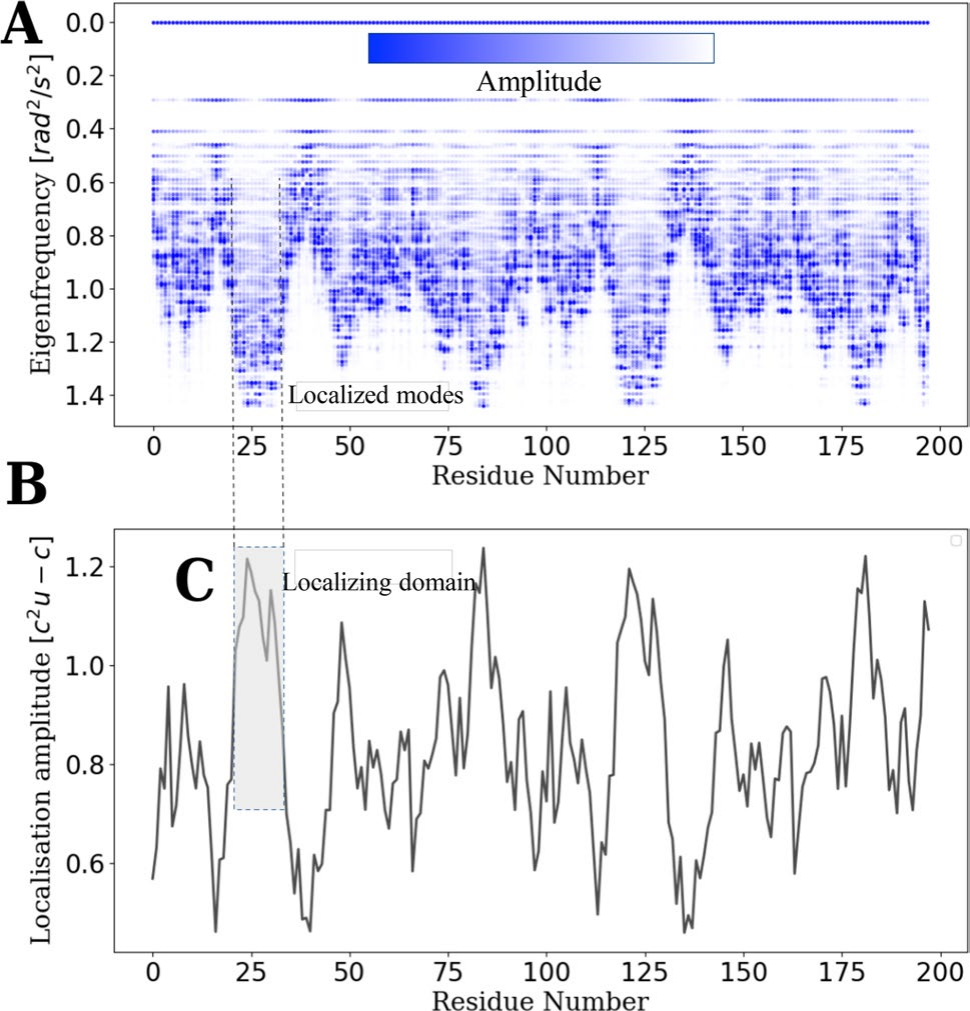
Backbone vibrations in HIV-1 protease. The spectrum of normal modes (**A**) reveals that high-frequency motions are concentrated within localizing domains termed ‘hot spots’. The localization landscape (**B**) reveals where the fastest vibrations are located by contouring these hot-spots along the residue sequence (**C**).

$x_{m/n}$ stands for the coordinate of the AA along a chain of length *N*. A real constant *c* is introduced such that the eigenvalues of $\Delta _h > 0$. In practice, *c* can be taken slightly greater than the greatest eigenvalue $\omega _{max}^2$ of $\Delta _d$. Since the periodicity of the AA chain is $a=0.38$ nm, it is important to consider the wavevector $k=\pi /a$. The localization amplitude along the AA chain at a position *i* can be thus expresses as

10 $$\begin{aligned} {{ u}_i} = \frac{{1 - \sum \limits _{j=1}^{N} {{\alpha _{ij}}} {{ u}_j}{{( - 1)}^{{n_{ij}}}}}}{{c - \sum \limits _{j=1}^{N} {{\alpha _{ij}}} }}, \end{aligned}$$

where $n_{ij}$ stands for the number of unit-cell of length *a* separating two residues *i* and *j* along the AA sequence. The ability of this quantity to identify where energy localizes can be assessed in Fig. 2.

The term $\sum\limits_{j \sim i} {{\alpha _{ij}}{u_j}{{( - 1)}^{{n_{ij}}}}}$ introduced in the previous expression converges rapidly with the sum as $\alpha _{ij}$ strongly decreases when increasing *j*, due to the nature of the effective potential that generally depends on ${\left| {{X_i} - {X_j}} \right|^{ - \alpha }}$, with $\alpha > 1$. In addition, Eq. (8) ensures that ${u_j} < 1,\forall j \in \left[ {1,N} \right]$. Hence, Eq. (10) approximates as,

11 $$\begin{aligned} {{{\tilde{u}}}_i} \approx \frac{{1 + \varepsilon }}{{c - \sum \limits _{j \sim i} {{\alpha _{ij}}} }}. \end{aligned}$$

Having $\varepsilon \ll 1$ and $c > \sum \limits _{j \sim i} {{\alpha _{ij}}}$, one can write

12 $$\begin{aligned} {u_i} \approx 1/c + 1/{c^2}\sum \limits _{j \sim i} {{\alpha _{ij}}} \end{aligned}$$

**Figure 3:**
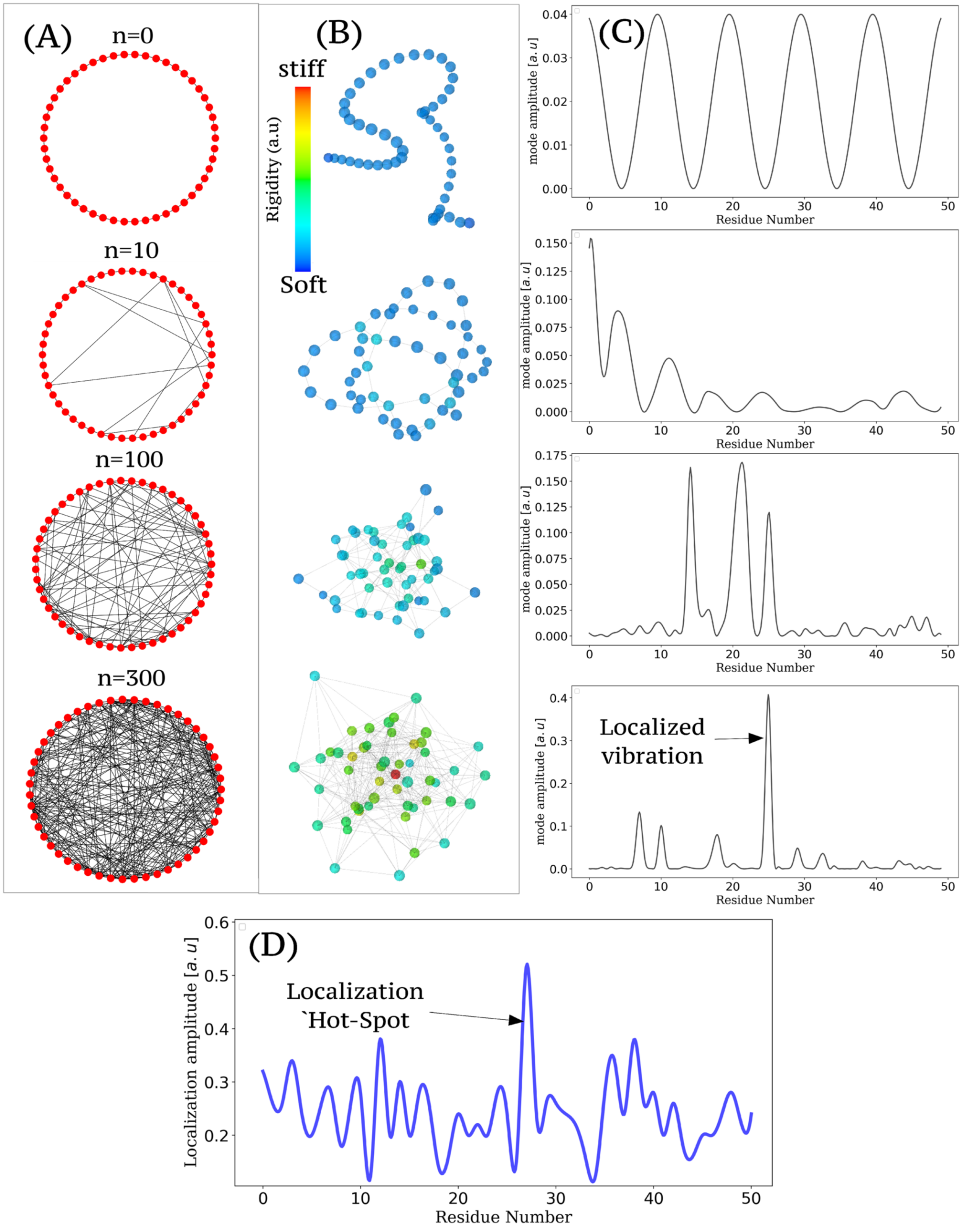
Native contacts, rigidity and the formation of a hot-spot. (**A**) Network representation of 10 to 300 native contacts, included randomly along a polymer chain made of 50 AA which consequently folds (**B**). The most rigid part are identified in red. The highest frequency mode (**C**) tends to localize at the hot-spot indicated by the localization landscape (**D**) and corresponding to the stiffest residue (red bead on **B**).

Interestingly, this important result states that thermal vibrations localizes in proteins as a linear function of the local rigidity of the backbone chain. Let’s illustrate this tool at work by considering a toy model of polymer chain as short as 50 AA in length. The folding into stable 3D structures has been mimicked by including random native contacts (10 to 300) as illustrated in Fig. 3. As this interaction parameter increases, the cohesion of the structure is reinforced, rigid parts get formed and finally a hot spots arises. Without native contacts, the spatial structure of the highest frequency mode delocalizes/extends over the entire polymer domain (the motions of all residues are mechanically coupled). In contrast, almost all of the system energy is localized at a localization hot-spot (residue 26) with less than an 3 random native contacts per AA in average.

**Figure 4:**
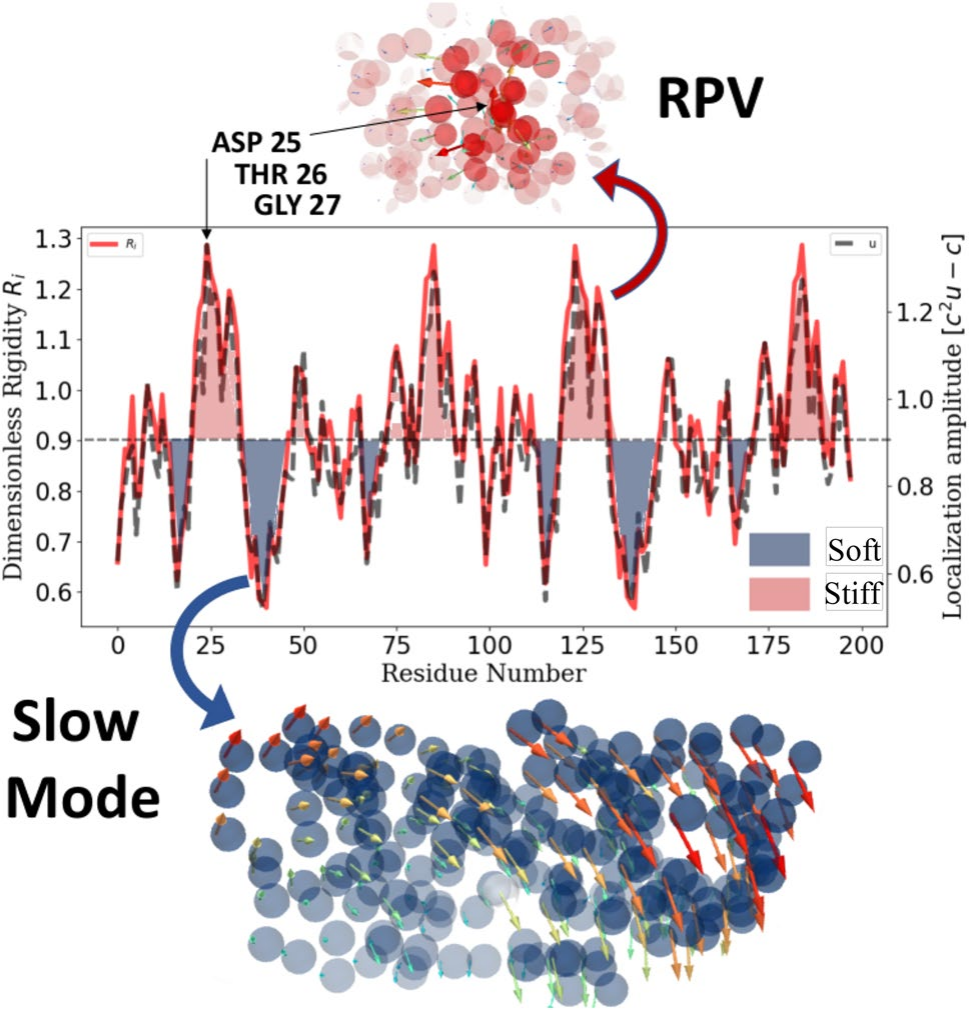
Rigidity pattern versus localization landscape The backbone structure can be considered as a discontinuous sequence of stiff and soft domains. Slow modes (change of conformation) are predicted by the soft regions which from the valleys of the LL (blue). High-frequency vibrations are found in the stiffer part of the backbone (red). A RPV corresponds to high frequency motion localized at the vicinity of the active sites.

**Figure 5:**
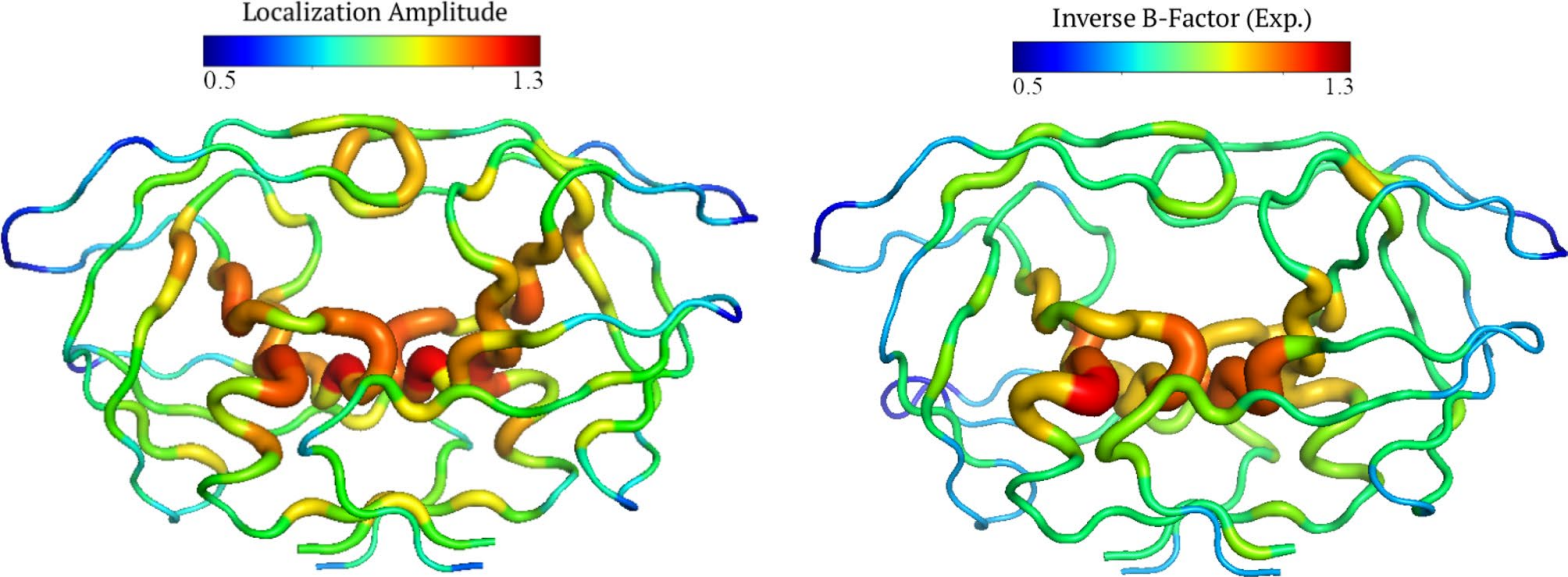
Comparison between the LL and the inverse temperature factor The stiffness of the backbone structure is a way to vizualize to the localization landscape (see Eq. (12)). This energy landscape corresponds to the inverse of the temperature factor (Eq. (7)). The CF between the two quantities reaches 0.76 in this case (PDB id: 1odw). The figures was rendered using PyMOL (The PyMOL Molecular Graphics System; http://www.pymol.org).

The correspondence between energy localization and the rigidity profile is quantitatively validated on Fig. 4 with a more realistic enzyme. In the case of HIV-1 protease, rigidity and localization correlate with a correlation factor $CF>0.9$. A 3D representation of the rigidity profile/localization landscape is depicted on Fig. 5.

By virtue of the equipartition theorem, at thermal equilibrium with a temperature *T*,

13 $$\begin{aligned} \sum \limits _{j \sim i} {{\alpha _{ij}}} \left\langle {X_i^2} \right\rangle = 3{k_B}T. \end{aligned}$$

Interestingly, the localization landscape and the inverse of the B-factor are linearly dependent:

14 $$\begin{aligned} {u_i} \approx a + bB_i^{-1} \end{aligned}$$

Equations (10) and (12) tells us that the localization landscape (i.e the local density of states) is completely driven by the rigidity profile along the BB chain. Eq. (14) implies that this quantity can be observed directly from the temperature factor.

To sum up, when discussing structural aspects, enzymes are not just ‘ordered’ or ‘disordered’ sequences of residues as it is commonly discussed in biophysics. The particular symmetry associated with the periodic chain of AA allows wave propagation to occur (i.e., a transport mechanism by conduction that diffuses thermal energy in the scaffold). From this consideration, a mathematical operator is constructed to detect whether this energy localizes or spread everywhere in the system. Wave localization is the particular feature where vibrations are spatially confined within domains much shorter than the full sequence of residues. This mathematical approach provides a quantitative criterion to identify functional residues associated with potential fast dynamical effects. Indeed, by analyzing the so-called localization landscape, we found that fast-vibrations are clamped at the stiffer part of the enzyme scaffold. More specifically, the localization amplitude is a linear function of the local rigidity, and rigidity modulation drives the characteristic timescales associated with the dynamics of each residue. Hence, to probe fast-dynamical effects in an enzyme, one must focus on the stiffer parts of its structure.

## Results

The energy localization landscape (LL) offers an accurate representation of how thermal energy of the BB is partitioned within the scaffold (see ex. Fig. 5). In other words, LL reveals hot-spots corresponding to strings of residues that are mechanically coupled, and in which most of the *THz* energy is trapped. An important finding in this work is the similitude between the BB rigidity and the energy (LL) of the protein structure (Eq. (12)). In order to demonstrate that the BB rigidity (Eq. (6)) dictates the pattern of localized vibrations (Eq. (10)), the two quantities have been calculated and compared in a systematic study on the catalytic site atlas (CSA)^27^, revealing an average correlation factor (CF) of 0.98 as exemplified in Figs. 4 and 5.

This planned comparison reveals first that, discerning the backbone rigidity profile allows one to precisely delineate where all of the fastest vibrations (i.e in the ps range) appear in the 3D scaffold (e.g., Fig. 4). The pattern of vibrations takes the form of the stiffness discontinuity resulting from a defined folded state. Interestingly, a consequence of free energy minimization is the formation of clusters of coupled/decoupled residues through the emergence of a new landscape. To further illustrate this substantial finding, we extracted the temperature factor (Eq. (6)). We compared it to the LL with a correlation factor (CF), indicating that there is $76\%$ of similarity between the energy and the rigidity distribution (see Fig. 4) for this specific structure (PDB id:1odw).

**Figure 6:**
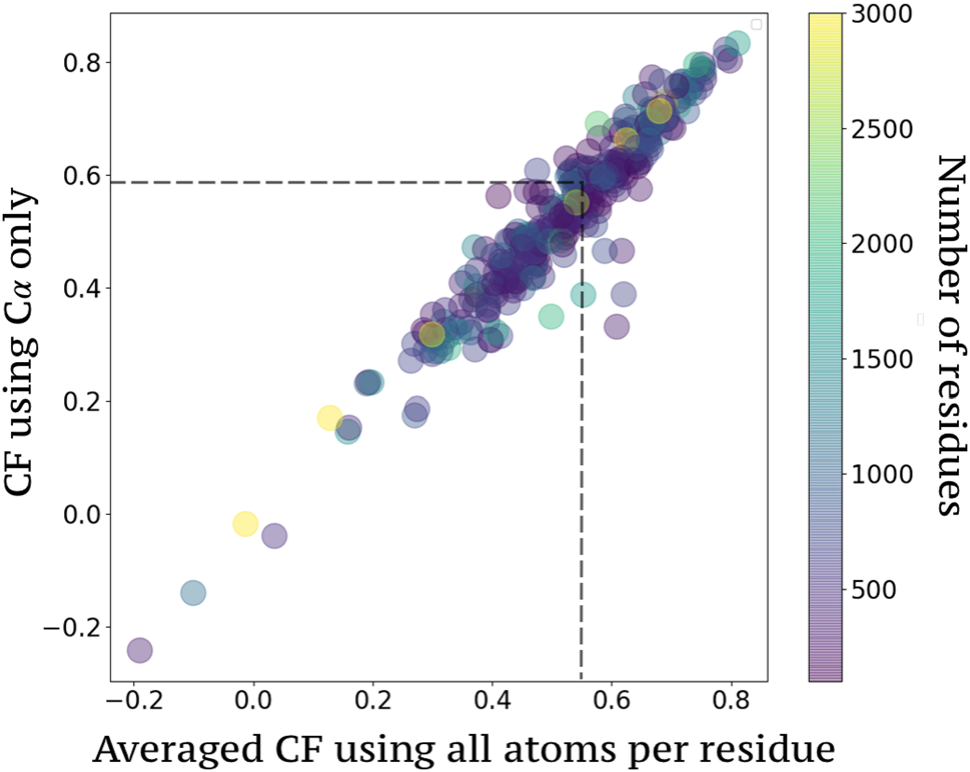
Correlation between the localization landscape and the inverse temperature factor. The stiffness of the backbone structures are extracted from the temperature factor (Eq. (11)) in the CSA database and compared to the localization landscape, revealing an average CF of 0.59).

**Figure 7:**
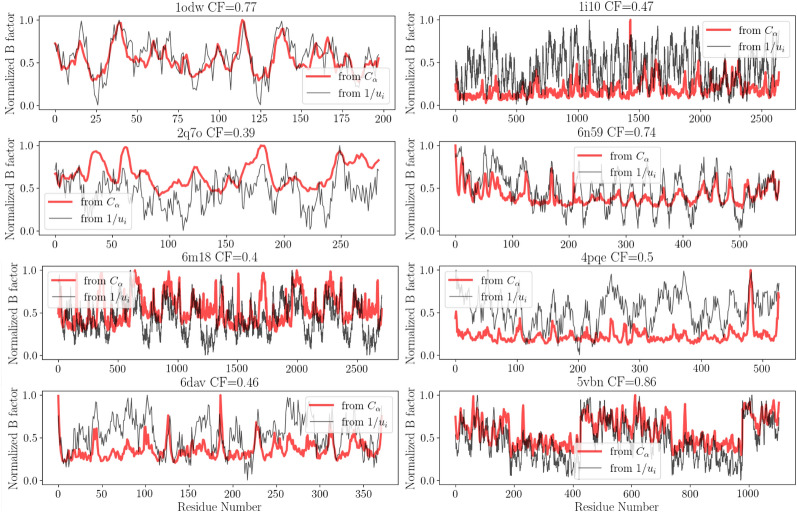
Temperature factor and localization landscape The B-factor is compared to the inverse localization landscape 1/*u* for HIV1-protease (PDB id: 1odw), DHFR (PDB id:6dav), LDH (PDB id: 1i10), Caspase (PDB id: 1i9w), ACE2 (PDB id: 6m18), [Fe Fe] Hydrogenase (PDB id: 6n59), acetylcholine (PDB id: 4pqe), PNP (PDB id: 2q7o), DNA polymerase epsilon B-subunit (PDB id: 5vbn).

To further corroborate the range of validity of this observation, this calculation has been conducted on the same statistical sample of enzymes mentioned earlier. An average correlation of 0.59 with a standard deviation of 0.13 (Fig. 6) has been found. Low correlation factors are independent of the protein sequence length (expressed here number of residues). Considering the relative simplicity of our model of interaction and several inaccuracies included in the experimental data, it demonstrates that this analysis contains a robust and accurate prediction level. Indeed, B-factors typically reflect not only intrinsic flexibility but also structural errors. They are affected by crystal packing, which can often artificially stabilize flexible areas (e.g., B-factors are artificially low) or even create disorder when incompatible regions are forced into proximity by the lattice arrangement. They are a result of static disorder within the crystal (neighboring molecules in the lattice in different orientations, mainly due to flash cooling at 100 K during data collection) and dynamic disorder (conformational differences throughout structure). Different structures of HIV-protease (PDB 1HPV, 1RX7, 1DMP, 7HVP, 1ODW) have been tested and it has been found found that according to the structural errors included in the diffraction experiments, the CF dramatically spread from 0.4 to 0.75 for the same topology and independently of the resolution cutoff. The very weak correlation scores have—to our knowledge—no direct association with a particular type of enzyme (example: IDP) but seem more associated with experimental conditions. A more detailed representation of the match between the B-factor and the localization landscape is proposed on a set of enzymes of interest, in Fig. 7. The more the B-factors is a reliable measure of the protein’s thermal motions, the better it allows a direct access of the LL. It therefore seems prudent to proceed to ensemble refinement^28^ for any future study highlighting the effects of energy localization, on the basis of an interpretation of the B-factor.

To a high degree, it has been observed that more than 80% of the BB energy is localized in enzymes. In other terms, for 603 modes describing the picosecond dynamics of HIV1-protease, more than 480 have a structure predicted by the LL (Fig. 2); Besides, most of the BB energy ’trapped’ within segments greater than 5 but smaller than 10 residues in length. Active sites (Asp25, Thr26, Gly27) are located in the stiffest parts (Fig. 4), with the highest energy density. This is physically consistent with the observation that roughly 80% of the catalytic residues are located at the hardest parts of enzyme structures^14,15,29^. The possible functional significance of the remaining 20% of residues associated with disordered domains is discussed later in this work. Interestingly, it has also been observed throughout this study that there is practically no distinctions between the localization landscapes of any structured protein and that of an enzyme (In the sense that the landscapes exhibit the same trends, with cols and valleys describing localization hot-spots ranging from 10 to 20 residues in length). In fact, if the presence of RVPs is a prerequisite for catalysis in enzyme then, all proteins are potentially enzymes. From now, the question asked in the introduction shall be re-expressed thus: How do we accommodate topologically the recognized importance of protein rigidity with the emergence of functional residues associated with ps dynamics? In doing so, can we provide a physical basis for the existence of RPVs?

**Figure 8:**
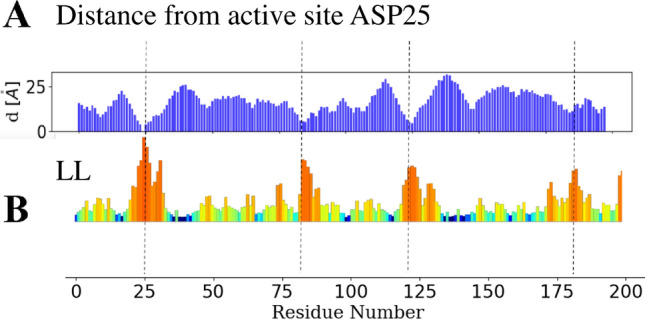
Cartesian distances between ASP25 and other residue compared to LL Hot spots which host RPVs are in general highly local (dash lines), they are not uniformly formed along the amino acid chain. Large distances in AA location can be very short in the 3D space, suggesting that multiple segments of the protein can be involved in the creation of RPVs.

An enzyme domain is said to be ordered if its hydropathy is enough to drive spontaneous folding. In this work, a complementary vision of the concept of order is introduced when studying ps dynamics. Indeed, thermal energy transport arises from the long-range ordering of the $C_\alpha$ of the BB that allows waves to propagate along the leading chains. When the protein folds, its residues form native contacts that brake this translation symmetry, and they do so by adding strength, which is commonly referred to as order. This break in the invariance of the BB stiffness or discontinuities, fold-encoded in the structure, concentrates the motions of the protein to specific ’hot spot’ residues. Such hub residues, because they are subjected to an increased density of fast (ps) compressive motions between atoms, have a better propensity to search for optimal configurations. Therefore they are likely to display a functional role in catalysis, such as hosting RPVs. The euclidian distances between the active site Asp25 and other residues (Fig. 8) have been computed and compared the profile obtained with the localization landscape (B).

While RPV’s (or at least hot-spots) are highly local (dash lines), they are not uniformly formed along the amino acid chain. Indeed, the cartesian distances between ASP25 and three of the four main hot-spots are shorter than 0.5 nm. This finding suggests that multiple segments of the protein, separated by vast distances in amino acid location (> 50 residues), can be involved in creating RPVs. Interestingly, this information is partially accessible from the observation of radiation attenuation (B-factor) in a diffraction experiment, provided that structural errors associated with crystal packing are limited.

### Interpretation

Our approach provides a physical rationale that explains the emergence of a biological function (the exploration to a more effective transition state barrier through thermal vibrations) as a tradeoff between order and disorder, which would be more appropriately termed continuity/discontinuity. Order/continuity allows energy to propagate, while disorder/discontinuity portions the structure into distinct strings of mechanically coupled residues, fed with an enhanced density of high (ps) frequency motions. Besides, a moderate correlation between our model and the temperature factor establishes the central role of the full topology in the THz regime. Although there is no explicit requirement for a universal theory of enzyme catalysis, one can extract the following features from the interpretation of the results.

- In enzymes, most of the energy coming from the backbone vibrations is confined within the most rigid parts of the structure. Active sites in enzymes, because they are usually stiff, are subjected to RPVs.
- The geography of hot-spots is defined by the shape of the folded protein.
- The localization landscape is a feature conserved throughout evolution.

These findings not only match the state of the art methods to understand the role of rigidity and the importance of order/disorder in enzyme-catalyzed reactions, but they also validate the debated physical origin of RPVs, which now appears as a solution to solving the problem of increasing the catalytic proficiency while maintaining the protein stability-function tradeoff using an available energy source arising from thermal fluctuations. As a by-product of this tradeoff, stiffness-driven localized vibrations provide a first glimpse into how the ps timescale was possibly selected and explain the molecular and physical origin of RPVs^7,9^.

## Discussion

Previous works have hypothesized that differentiation and low connectivity between a protein’s scaffold and its active-site is a crucial prerequisite for innovability^30^. Indeed, in light of the LL, differentiation and low connectivity produce a partitioning of the BB’s thermal energy, which induces the mechanical decoupling of all AA motions, a prior step to the establishment of functional residues. Many biologically active proteins do not need to have a unique fixed and rigid 3-D structure to execute a function^31,32^. Examples are the abundance and functional importance of intrinsically disordered protein (IDP) but not exclusively. Much than 44% of the human proteome contains intrinsically disordered peptide segments more significant than 30 residues in length^33^. Surprisingly, the majority of disordered peptide segments have yet no known function^34^. Though, if one considers protein dynamics from its vibrational energy property (rather than focusing on motions of significant variance during the conformational changes), disordered regions could be a prerequisite for allosteric pathways: By damping mechanical waves, they promote coupling between specific residue. Therefore, an additional by-product associated with the rigidity would allow solving the problem of regulation with an evolutionary strategy that consists in modulating to the pattern of mechanical damping, such that signals propagate between selected groups of distal residues.

Hence, from this study, it appeared that energy/information transport requires, on the one hand, long-range symmetry to emerge from random thermal fluctuations. To take advantage of this available energy in a functional form, folding produce discontinuities of the scaffold rigidity that comes to dictates the configuration of energetic hot-spots, or possible functional residues. These hot-spots fuels the enzymes with potential RPVs and would eventually become active sites throughout the natural selection. Putting forward this analysis in the line of a canonical Darwinian vision, the energy localization pattern—as much as its counterpart, the free energy landscape—appears as an essential (albeit elegant) attribute of enzymes evolution extensible to all proteins in general. Hence, the conventional paradigm of the enzyme structure that encodes the function is prolonged here to the structure that encodes the dynamics and the catalytic rate acceleration. Together, both structure and dynamics encode the biological function. We finally propose a new class of experiments that can be achieved from the basis of this theory.

**Figure 9:**
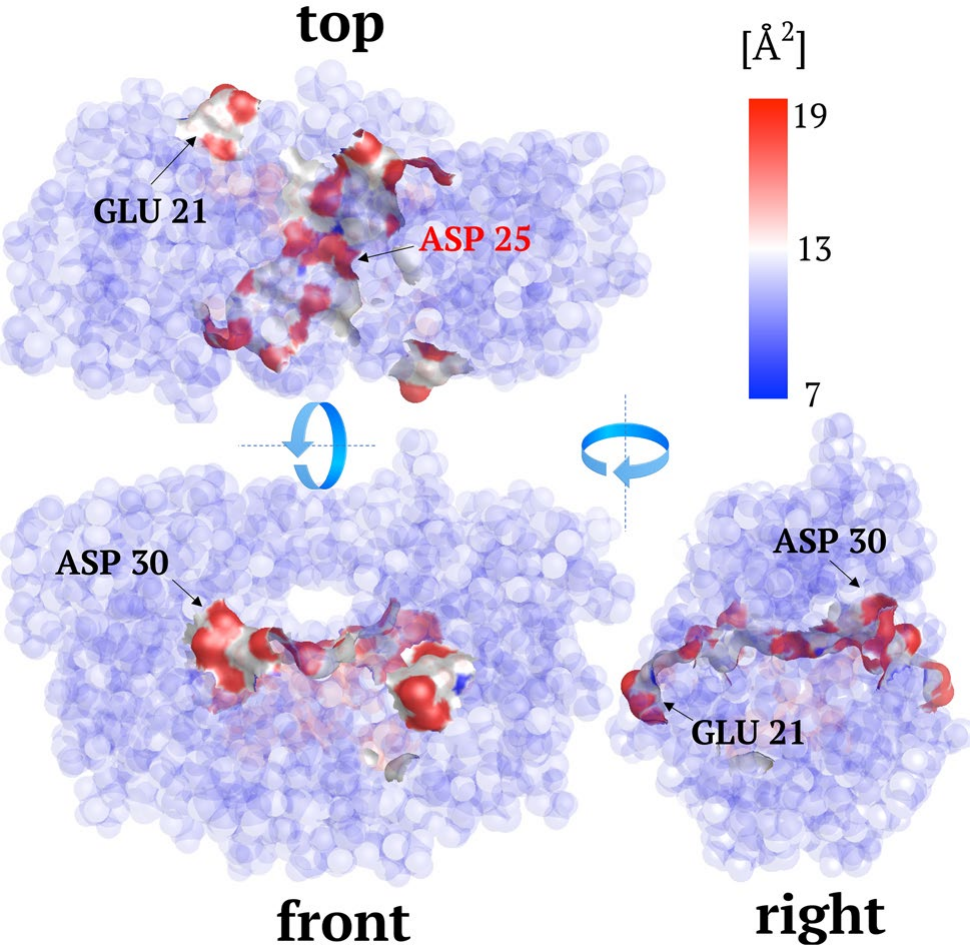
Binding sites influencing the dynamics at active sites in HIV-1 protease. The position of hot spot residues are correlated to these having a high solvent accessible surface area in order to detect potent bonding sites that dynamically influence the catalytic sites. The figure was rendered using PyMOL (The PyMOL Molecular Graphics System; http://www.pymol.org).

1. To determine the ideal sites for a small-molecule to bind and influence the catalytic rate, one first extracts the coordinates of the localization hot-spots (i.e. their positions along the sequence and waists). By correlating these strings of hot-spot residues to a measure of the solvent’s accessibility, one can predict potential candidates (residues) that will affect the catalytic rate upon binding. We tested the possibility of this instrument on active sites residues Asp25Thr26Gly27 located on the localization hot spot corresponding to residues ranging from 21 to 31. Potential binding site candidates such as GLU21 as well as ASP30 have been extracted (Fig. 9).
2. Considering the extremely light computational costs of this rigidity-based LL, it is possible to investigate almost instantaneously the effect of distal mutations on the dynamical properties of catalytic sites. Such a direct approach to modulate RPVs’ effects allows narrowing the results of large-scale screenings considerably.

However, since this work is currently based on ENM, there are several limits, notably regarding the possibility to investigate the influence of co-factors on the LL. Additional investigations are currently conducted to extend this dynamical analysis using more realistic force fields with MD simulations to produce more accurate dynamical matrix.

## Conclusion

In this paper, we have introduced an atomistic and robust structure-based approach that identifies the structural rigidity of the BB as a key quantity to understand and control RPVs at catalytic sites. Our results clarify the deep connection between an enzyme structure, the rigidity arising from the molecular interactions, and the subsequent picosecond dynamics that emerge to directly modulate the catalytic rate at active sites in a fold-encoded fashion. Hence, in enzymes, the observation of discontinuity of the BB stiffness provides a unified picture of how the search time to actives configurations splits into two distinct but complementary dynamical scales: slow conformation changes by soft domains, and fast promoting vibrations occurring at the stiffest parts. RPVs or fast dynamical effects appear as a by-product of the stability-function tradeoff in enzymes and possibly in proteins in general. This molecular theory paves the way toward a rational design of catalytic proteins and provides simple conceptual tools to guide experiments addressing the influence of vibrations on chemistry.
